# Impact of Emotional Intelligence on Professional Performance and Stress Resilience Among Healthcare Practitioners

**DOI:** 10.7759/cureus.74113

**Published:** 2024-11-20

**Authors:** Sherbano Hashmi, Okasha Tahir, Zeeshan Nasir, Haider Hasnain, Raja Saif Ullah Riaz, Hafiz Muhammad Ali Khan, Rizwan Ramza, Hamzah M Alghzawi, Jina Mukherjee, Anvi Palav

**Affiliations:** 1 Department of Clinical Psychology, Shifa Tameer-E-Millat University, Islamabad, PAK; 2 Department of Medicine, Bacha Khan Medical College, Mardan, PAK; 3 Department of Medicine, Nishtar Medical University, Multan, PAK; 4 Department of Medicine, Federal Medical College, Islamabad, PAK; 5 Department of Health Sciences, National University Science and Technology, Islamabad, PAK; 6 Deparment of Internal Medicine, East Lancashire Hospitals National Health Serrvice (NHS) Trust, London, GBR; 7 School of Nursing, Tennessee State University, Nashville, USA; 8 Science, G.D. Goenka Public School, Dakshineswar, IND; 9 Biological Sciences, Ridge High School, Basking Ridge, USA

**Keywords:** coping ability, emotional intelligence, healthcare professionals, job performance, stress resilience

## Abstract

Objectives

Emotional intelligence (EI) refers to the ability to perceive, understand, and manage emotions effectively, a skill essential in the high-stress environment of healthcare. Research suggests that healthcare professionals with higher EI are better equipped to handle stress, maintain resilience, and make sound judgments under pressure, ultimately enhancing job performance. This paper examines EI’s predictive role in managing job performance and resistance to stress among healthcare professionals, aiming to explore how elevated EI may strengthen their coping abilities and contribute to improved stress management, professional judgment, and resilience in challenging work settings.

Method

This cross-sectional study was conducted among 108 healthcare providers, including doctors, nurses, and allied workers. It used validated questionnaires to collect data using the Trait Emotional Intelligence Questionnaire (TEIQue) to measure EI, the Perceived Stress Scale (PSS) to determine the level of stress, and other standardized tools to grade job performance and the level of resilience. SPSS analyzed data to find the correlation between EI, stress level, job performance, and resilience. IBM SPSS Statistics, Version 26 (IBM Corp., Armonk, NY, USA), was used to analyze data and determine the correlation between EI, stress level, job performance, and resilience.

Results

The results showed a high positive correlation between EI and job performance, r = 0.601 and resilience r = 0.626, suggesting that higher levels of EI positively determine effective professional outcomes and greater resilience. Besides, EI was shown to have a moderate positive correlation with stress management, r = 0.624, indicating that higher EI levels enhance the capabilities of health professionals to manage stress effectively.

Conclusion

This research would establish that EI significantly affects the resilience of healthcare professionals to stress and job performance. This means that EI-enhancing programs implemented in healthcare agencies could result in better health outcomes, low burnout, and higher resilience of healthcare workers. The subsequent studies may examine the long-term outcomes of EI on resilience to stress and job performance in various settings.

## Introduction

Healthcare providers work in high-stakes, high-pressure environments with hundreds of stressors, ranging from overcrowded organizations and suboptimal equipment to difficult working conditions. Some impacts are on the psyche, but others directly affect patients' quality of care, lowering the quality of both work and lifetime for practitioners. Apart from situational stressors, emotional stressors such as fear and anxiety may impact problem-solving capacity, which is crucially required for healthcare provision intended to protect public health and quality of life [1].

Burnout among healthcare personnel is very high, and interpersonal reasons within the health field intensify competition, thus aggravating burnout. Stress levels were already alarmingly high among health professionals even before the COVID-19 pandemic, with rates over 0.60 (60%); recent studies indicate that these levels have now risen above 0.70 (70%) [2]. There are many challenges for healthcare workers such as work overload, lack of resources, or high emotional demands which have a negative impact on their mental health and patients. A study points out that a lack of work-life balance is one of the biggest sources of these problems since it worsens stress levels and results in greater burnout rates among practitioners [3].

The fact that these challenges are related to healthcare has led to the development of emotional intelligence (EI), which has surfaced as a significant determinant of success in healthcare. This enables the various workplace staff to manage conflicts and high-stress environments involving complex interpersonal interactions. EI is recognizing and understanding one's emotions and those of others [4,5]. This capability is considered to be of much importance in the healthcare setting, given the fact that decisions may affect patient outcomes and well-being [6]. Studies indicate that more emotionally intelligent professionals contribute to leading, teamwork, increased patient outcomes, and lower staff turnover, particularly among nursing professionals and healthcare management [7,8]. Emotionally intelligent practitioners tend to do better in practicing and fulfilling their duties to the patient, creating teamwork, and reducing workplace conflict.

Studies reveal that when the EI levels are discovered to be high, the workforce promotes the causes of ethical nursing practices. Professionals with higher levels of EI are at ease when handling emotional stressors in patient communication. They are not hesitant to make tough choices while exercising ethical decisions. Emotional resilience takes center stage in this regard because it is critical in effectively handling patient care and achieving professional standards in hostile circumstances [9].

While the nursing domain has universally acknowledged the benefits of EI, the impact of EI across other healthcare professions needs to be more researched. This paper addresses the lacuna by discussing the relationship of EI with professional functioning across different disciplines of healthcare practice. It explicitly considers how EI influences decision-making, patient communication, teamwork in clinical environments, and experiences of work-related stress and burnout. These study results are likely strong in showing how this relevant skill set contributes to stress resilience among healthcare professionals.

Besides serving to enhance professional performance, EI is related to stress resilience. Healthcare practitioners are subject to emotionally challenging situations like patient suffering, long working hours, and high expectations [10]. Research has shown that individuals with excellent EI better manage stress and prevent burnout [11], which results in higher job satisfaction and more excellent professional retention [12]. EI can also be a moderating variable by generating positive coping mechanisms such as problem-focused strategies instead of avoidance. Healthcare practitioners with these competencies will probably secure their emotional welfare in pressured situations [13].

Knowledge of the interplay between EI, professional performance, and resilience to stress might be critical for designing the interventions and the training program that target the healthcare environment's betterment [14]. Thus, the study intends to examine the relationship between EI, professional performance, and stress resilience in healthcare practitioners. In a context that aims to contribute to the growing body of evidence supporting the inclusion of EI training in professional development programs for healthcare workers, it discusses the relationship between EI and GP care team performance. It then undertakes some exploratory work that goes beyond this aim regarding how EI could support human resources as a buffer of workplace stress, facilitating a healthier environment at work and for those cared for afterward.

Rationale

Those in the health sector will often need to make urgent decisions, use excellent interpersonal communication, and be responsible for their emotions in a constantly stressful condition. Thus, under such conditions, EI is becoming crucial as a performance and resistance factor for healthcare practitioners. It is understood as the ability to observe, acknowledge, and deal with one's emotions and the emotions of others in such a way that it plays a critical role in the augmentation of the situation at workplaces with optimum care for the patient.

Health professionals, that is, physicians, dentists, nurses, and allied health professionals, are exposed to very emotive situations while treating their patients. This may range from patients suffering from complex family matters to critical emergency conditions. Highly emotionally intelligent people deal with the patient's sensitive situations with empathy, calmness, and appropriate collaboration between a patient and his peers. Besides the above, EI develops problem-solving ability and improves communication, thus creating better management with fewer errors.

Another significant link has been established between EI and stress resilience. The better a practitioner will handle job-related stress and avoid burnout, the higher a practitioner's EI score and greater psychological well-being. Healthcare consists of heavy workloads, long hours, and the emotional toll of patient care, which indicates a high prevalence of burnout and stress. It sometimes triggers a chain reaction of mental health issues among the healthcare providers. The theory of EI may act as a protection factor in this respect. This will ensure that job satisfaction and performance are kept at extremely high levels, even in the most stressful situations, and prevent stress from spilling out of proportion.

With increasing awareness that EI plays an essential role in competency among health professionals, this study seeks to ascertain whether EI impacts performance at work and stress resilience among healthcare professionals. This information is, therefore, crucial in guiding the design of training programs that can focus on EI improvement, leading to better patient outcomes, reduced burnout among practitioners, and other aspects of better, more sustainable healthcare workforces.

The research is comparatively timely and relevant, especially to the growing pressure on most healthcare workers, for instance, as exemplified by the COVID-19 pandemic that has spread every last inch of the world, triggering so much increased stress among stakeholders and heaping unbearable pressure on them.

## Materials and methods

We investigated the connection between EI, professional performance, and stress resilience among healthcare professionals through a quantitative, cross-sectional study across various hospitals and clinical settings. The study population encompasses healthcare providers such as doctors, nurses, allied health staff, and all other types of clinical workers over 18 years who consented to this research. The inclusion criteria included subjects above 18 who were working in any healthcare setting. The exclusion criteria consisted of healthcare professionals who were not actively working in a clinical setting, those under 18 years of age, and individuals who did not provide informed consent. Data collection was done through online questionnaires from July 2024 to September 2024.

Sample and sampling technique

The sample size was calculated using the WHO sample size calculator at a 95% confidence interval and an expected population proportion of 0.80. However, due to time and resource constraints, this study was carried out with 108 participants from different professions and positions in the health sector through purposive sampling.

The trait Emotional Intelligence Questionnaire Short-form by Petrides and Furnham (2006) evaluated participants’ EI (Appendix A) [15]. It has 30 items measuring the accepted dimensions, including self-awareness, emotional regulation, and empathy [15]. The Perceived Stress Scale was developed by Cohen et al. (1983), and it has 10 items (Appendix B) to measure the perceived stress among the participants over the last month [16]. An adaptation of the Job Performance Scale from Campbell (1990), 40 items assess participants’ level of resilience (Appendix C), as it yields a high score with an outstanding level of resilience [17].

Moreover, the Resilience Scale of Connor and Davidson (2003) comprised 25 items applied in this research (Appendix D) [18]. It measures the participant’s ability to withstand stress and adversity [18]. All of the instruments used in the research were validated using Cronbach’s alpha and other reliability thresholds that exceeded tolerable levels of acceptable scores. The participants’ responses were recorded based on the Likert scales customized for every instrument utilized during the implementation stage.

Ethical clearance

The data were analyzed using IBM SPSS Statistics, Version 26 (IBM Corp., Armonk, NY, USA). Brain Tech Clinic and Research Center, Islamabad, which operates as an independent research organization with its own Institutional Review Board (IRB) provided ethical approval (IRB-2024-0015). The purpose of the study was communicated to the participants, and privacy and identity, as well as respecting their self-esteem, were preserved throughout. The data collected was anonymous and was used solely for academic work.

## Results

Table 1 shows the demographic characteristics of the participants. In terms of gender, the majority were male subjects, with 56 (51%) participants, while 52 (48%) were female subjects. Regarding education, 75 (69.9%) participants held a Bachelor's degree, followed by 14 (13%) with a Master's degree, and 19 (17%) with postgraduate qualifications. Occupationally, the sample included 46 (42%) medical doctors, 29 (26%) dentists, 14 (13%) allied health professionals, 10 (9%) health administrators, and nine (8%) participants in other professions. In terms of age, most participants were early adults, with 93 (86%) in this category, while 10 (9.3%) were middle adults, and five (4.1%) were old adults.

**Table 1:** Demographic characteristics of study participants. F = Frequency, % = Percentage.

Variable	F	%
Gender	-	-
Male	56	51
Female	52	48
Education	-	-
Bachelor’s degree	75	69.9
Master’s degree	14	13
Postgraduation	19	17
Occupation	-	-
Medical doctors	46	42
Dentists	29	26
Allied health professionals	14	13
Health administrator	10	9
Others	9	8
Age	-	-
Early adults	93	86
Middle adults	10	9.3
Old adults	5	4.1

Table 2 shows EI has a positive significant correlation with stress (r = 0.624), job performance (r = 0.601), and resilience (r = 0.626) in such a way that the higher the EI, the more the job performance and resilience, but also the stress levels. Similarly, stress correlates with job performance (r = 0.548) and resilience (r = 0.457). Interestingly, the variables job performance and resilience have the highest correlation (r = 0.755), demonstrating their interaction, and all the correlations are significant at 0.01 levels.

**Table 2:** Intercorrelation matrix of EI, stress, job performance, and resilience. EI = Emotional intelligence. ** = p<0.01 considered significant.

Variable	Emotional intelligence	Stress	Job performance	Resilience
Emotional intelligence	-	-	-	-
Stress	0.624**	-	-	-
Job performance	0.601**	0.548**	-	-
Resilience	0.626**	0.457**	0.755**	-

Table 3 shows no statistically significant differences among genders regarding EI, stress, job performance, or resilience, as all p-values (Sig. 2-tailed) are greater than 0.05. Specifically, EI (p = 0.525), stress (p = 0.237), job performance (p = 0.677), and resilience (p = 0.907) all fail to reach significance. As measured by Cohen's d, the effect sizes are small across all variables, suggesting that any observed differences between the groups are minimal and likely not meaningful in practical terms.

**Table 3:** Comparison of EI, stress, job performance, and resilience by gender. EI = Emotional intelligence, M = Mean, SD = Standard deviation, LL = Lower limit, UL = Upper limit. p<0.05 considered significant calculated by t test.

Variable	Male		Female		t	P	CI 95%		Cohen’s d
-	M	SD	M	SD	-		LL	UL	-
Emotional intelligence	123.7	21.7	126.8	27.9	-0.6	0.525	-12.6	6.9	0.12
Stress	21.3	6.1	22.7	6.5	-1.190	0.237	-3.8	0.9	0.23
Job performance	131.2	23.7	129.3	23.7	0.418	0.677	-7.1	10.9	0.08
Resilience	83.8	14.7	83.5	16.9	0.117	0.907	-5.1	5.8	0.02

Table 4 shows the comparison of variables by age revealing significant insights into their relationships. Stress levels show a statistically significant difference across age groups (p = 0.002) with a moderate to large effect size (η² = 0.367). This suggests that age influences stress significantly. In contrast, EI does not demonstrate a significant difference (p = 0.680), indicating that age may not impact this variable. Although job performance (p = 0.072) and resilience (p = 0.063) show trends toward significance, they do not reach conventional significance thresholds, suggesting that differences in these areas may not be strongly influenced by age. Overall, age appears to have a notable effect on stress but has less influence on EI, job performance, and resilience.

**Table 4:** Comparison of EI, stress, job performance, and resilience across age groups. EI = Emotional intelligence, M = Mean, SD = Standard deviation, df = Degrees of freedom, Significance = p-value, η² = effect size.

Variable	Mean ± SD	Df	F	Sign.	η2
Emotional intelligence	125.2 ± 24.8	107	1.6	0.06	0.27
Stress	22.0 ± 6.3	107	2.5	0.00	0.36
Job performance	130.3 ± 23.6	107	1.6	0.07	0.26
Resilience	83.7 ± 15.7	107	1.6	0.06	0.27

Table 5 shows the comparison of variables by gender indicating that none of the measured variables show statistically significant differences. For EI, the F-value is 0.414, with a significance level of 0.522, suggesting that gender does not significantly affect EI. Similarly, stress levels (F = 1.417, p = 0.237), job performance (F = 0.175, p = 0.677), and resilience (F = 0.014, p = 0.907) also demonstrate non-significant differences across genders, with all p-values exceeding the conventional threshold of 0.05. The effect sizes (η²) are minimal, ranging from 0.0001 to 0.0132, indicating that gender has a negligible impact on these variables. Overall, the results suggest that gender does not significantly influence EI, stress, job performance, or resilience.

**Table 5:** Comparison of EI, stress, job performance, and resilience by gender. EI = Emotional intelligence, M = Mean, SD = Standard deviation, df = Degrees of freedom, Significance = p-value, η² = effect size.

Variable	Mean ± SD	df	F	Sign.	η2
Emotional intelligence	125.2 ± 24.8	107	0.414	0.52	0.00
Stress	22.0 ± 6.3	107	1.417	0.23	0.01
Job performance	130.3 ± 23.6	107	0.175	0.67	0.00
Resilience	83.7 ± 15.7	107	0.014	0.90	0.00

Table 6 shows the comparison of variables by experience revealing that none of the measured variables exhibit statistically significant differences based on experience. EI shows an F-value of 0.768 with a significance level of 0.467, indicating that experience does not significantly influence this variable. Stress levels have an F-value of 2.625 and a p-value of 0.077, suggesting a trend toward significance but not reaching the conventional threshold of 0.05. Job performance (F = 0.616, p = 0.542) and resilience (F = 0.059, p = 0.943) also demonstrate non-significant differences. The effect sizes (η²) for all variables are small, ranging from 0.0011 to 0.0476, which indicates a negligible impact of experience on EI, stress, job performance, and resilience. Overall, these results suggest that experience does not play a significant role in influencing these psychological variables.

**Table 6:** Comparison of EI, stress, job performance, and resilience by experience. EI = Emotional intelligence, M = Mean, SD = Standard deviation, df = Degrees of freedom, Significance = p-value, η² = effect size.

Variable	Mean ± SD	df	F	p	η2
Emotional intelligence	125.2 ± 24.8	107	0.76	0.46	0.01
Stress	22.0 ± 6.3	107	2.62	0.07	0.04
Job performance	130.3 ± 23.6	107	0.61	0.54	0.01
Resilience	83.7 ± 15.7	107	0.05	0.94	0.00

Table 7 shows the comparison of variables by occupation indicating no statistically significant differences among the measured variables. EI shows an F-value of 0.681 with a significance level of 0.606, suggesting that occupation does not significantly impact this variable. Similarly, stress levels (F = 0.955, p = 0.436) and job performance (F = 1.479, p = 0.214) also reflect non-significant differences across occupations. Resilience presents an F-value of 1.404 with a p-value of 0.238, further confirming the lack of significant effects. The effect sizes (η²) for all variables are small, ranging from 0.0258 to 0.0543, indicating a negligible impact of occupation on EI, stress, job performance, and resilience. Overall, these results suggest that occupation does not play a significant role in influencing these psychological variables.

**Table 7:** Comparison of EI, stress, job performance, and resilience by occupation. EI = Emotional intelligence, M = Mean, SD = Standard deviation, df = Degrees of freedom, Significance = p-value, η² = effect size.

Variable	Mean ± SD	Df	F	p	η2
Emotional intelligence	125.2 ± 24.8	107	0.68	0.60	0.02
Stress	22.0 ± 6.3	107	0.95	0.43	0.03
Job performance	130.3 ± 23.6	107	1.47	0.21	0.05
Resilience	83.7 ± 15.7	107	1.40	0.23	0.05

## Discussion

Most agree that work in the healthcare sector represents a highly high-stress area with workloads, intense emotional demands, and a constant need for daily patient care. Such factors strongly affect the mental state of healthcare workers, mainly resulting in burnout, job dissatisfaction, or unaccomplished performance. Scientific studies have indicated that inadequate staffing, insufficient resources, and poor working conditions aggravate these factors, leading to a hostile environment at work that affects not only healthcare providers but also patient outcomes [19].

The professional healthcare worker must always deal with difficult situations that must be solved within seconds, often without actual control of emotions or being actively engaged with others. In this regard, the importance of high EI is drastically underlined as a significant component of healthcare professionals' work effectiveness and ability to manage stressful events. EI refers to recognizing and understanding feelings of self and others. This essential skill is critical to a healthy working climate and excellent patient health care provision.

The findings of this study underscore the need for EI in the healthcare sector, which reduces stress and maximizes work performance across various ranges of health employees. The intercorrelation analysis indicated that EI positively correlates significantly with stress, r = 0.624, p < 0.01; job performance, r = 0.601, p < 0.01; and resilience, r = 0.626, p < 0.01. The results suggest that high EI levels are associated with high job performance, resilience, and stress. Studies have been conducted, and enough evidence has been shown to exist between EI and the provision of quality patient care, as published earlier [20].

Results in this study reveal that high EI leads to good problem-solving skills, effective communication, and regulation of emotions related to patient care management. All these skills are essential while working in stressful environments, combined with burnout and mental exhaustion. Therefore, the close association between EI and burnout resilience is a strong case for the argument that EI may work as a buffer for chronic stress, especially in healthcare. This finding agrees with earlier research showing that professionals in healthcare who have high EI perform better at emotional labor, which directly reduces the possibility of burnout [21].

Since burnout among health workers has personal effects and ripples on patient outcomes, developing EI in practitioners may act as a buffer against this widespread phenomenon. As such, EI would improve the retention of health professionals- an essential factor since the population is aging and there is increased demand for younger doctors.

Our study indicates that the ethical behavior of healthcare professionals has a positive relationship with EI. The study ensures the generalizability of the relationship between the different healthcare professions and will ensure that earlier studies only apply to nurses. Professionals with high EI enable performances in meetings with patients and enact ethical decisions during periods of stress. The current study was designed to examine the relationship between EI, stress, job performance, and resilience of healthcare professionals. According to the results, these variables are highly interrelated, especially with the significant relationship between EI and job performance and resilience. Neither gender, age, profession, level of experience, nor education were relevant factors regarding EI, stress, job performance, or levels of resilience against expectations. The findings, therefore, demonstrate the dynamics of the emotional and psychological aspects of the healthcare profession and underline its complexity.

Significantly, the research study suggests a highly positive correlation between EI, job performance (r = 0.601), and resilience (r = 0.626). These results are strongly supported by other similar sources suggesting higher EI levels facilitate improved performance in interactions, problem-solving, and handling stress conditions [22]. Skills regarding consciousness, knowledge, and regulation of one's and other's emotions will facilitate effective management of complex states of emotions in health care providers to maintain appropriate relationships with patients and, therefore, better outcomes [23].

EI was positively related to stress (r = 0.624), job performance (r = 0.601), and resilience (r = 0.626). This indicates that the higher their EI levels, the better their working ability and the more stress resilience levels they have while at work. The other studies focus on EI's role in performing good jobs and stress-coping vocations as critical as healthcare [5,24]. The highly significant correlation between job performance and resilience, with r = 0.755, confirms that resilience is the backbone of ensuring that high levels of performance are maintained even under stress, as supported by earlier work concerning the role of resilience in healthcare settings [25].

The study also manifests a moderate positive correlation between stress and job performance (r = 0.548) and resilience (r = 0.457). Previous research demonstrated that chronic stress damages healthcare professionals' lives, worsens their condition, and decreases Job satisfaction [26]. A solid relationship between resilience and job performance (r = 0.755) puts resilience as the most crucial ingredient while obtaining steady consistency in performance under conditions of extreme levels of stress. However, the results indicate an explicit requirement in that, regarding the support of practitioners' effectiveness in coping with challenging conditions, there is an apparent need for interventions explicitly designed to build their resilience.

Notably, there was no statistically significant difference in EI, stress, resilience, or performance at work related to gender, experience, occupation, or level of education [27]. This is very important because these demographic features are widely believed to relate to psychological outcomes in the hospital. The lack of such findings makes EI a global competence among healthcare professionals, irrespective of the demographic feature.

However, age analysis suggests a significant variation in stress levels (p = 0.002). Age may influence the way it interprets these professionals to understand stress. Some specific demands of work-life commitments and emotional expenditure of patient care may be particularly challenging for young healthcare practitioners, primarily those under the Generation Z category. This change in generational expectations over work-life balance may involve specific interventions for younger practitioners to sail through their challenging careers in health [28].

This study's implications, on the contrary, demonstrate the general necessity for healthcare organizations that provide healthcare services to invest resources in EI development programs that would assist healthcare professionals in enhancing their job performance and resilience. By concentrating on increasing the levels of EI among the staff, such organizations help their employees cope effectively with the usual higher levels of stress that the job entails, thus ensuring better practitioner health and patient outcomes. On the other hand, since there are no significant socio-demographic variations, EI training is practical for all members of this organization, and there is no need to argue how useful it is for every employee.

Limitations of the study

However, the study has some limitations. Another limitation of this study is that although high EI is associated with better performance and resilience, it is also associated with susceptibility to stress, resulting in higher awareness of stressors. This raises the question of whether it suffices to reduce the stress burden on healthcare practitioners using EI training alone. The study also focused on short-term assessment focusing on a one-day boost of resources and why the resources were insufficient to withstand role stress. The sustained management of job performance over time was mainly overlooked. Understanding which approaches are appropriate for managing stress, which would vary across different healthcare roles, still needs to be established. More studies need to be done to fill these gaps and determine and assess the effects of time in monitoring and controlling the interventions made.

## Conclusions

In conclusion, emotional intelligence (EI) significantly enhances job performance and resilience among healthcare professionals, supporting their ability to manage stress effectively in high-pressure environments. The study’s findings demonstrate that higher EI is associated with better job performance, increased resilience, and improved stress management, highlighting the importance of EI as a key factor in healthcare settings. These results suggest that standardizing EI training in healthcare education and professional development could reduce burnout, increase job satisfaction, and ultimately improve patient care, contributing to a more resilient and capable healthcare workforce.

## Appendices

Appendix A

**Table 8:** Trait Emotional Intelligence Questionnaire-Short-Form (TEIQue-SF). The Emotional Intelligence Questionnaire Short-Form by Petrides and Furnham (2006) evaluated participants’ EI [15].

Items	Response
Expressing my emotions with words is not a problem for me.	-
I often find it difficult to see things from another person’s viewpoint.	-
On the whole, I am a highly motivated person.	-
I usually find it difficult to regulate my emotions.	-
I generally don’t find life enjoyable.	-
I can deal effectively with people.	-
I tend to change my mind frequently.	-
Many times, I can’t figure out what emotion I’m feeling.	-
I feel that I have a number of good qualities.	-
I often find it difficult to stand up for my rights.	-
I’m usually able to influence the way other people feel.	-
On the whole, I have a gloomy perspective on most things.	-
Those close to me often complain that I don’t treat them right.	-
I often find it difficult to adjust my life according to the circumstances.	-
On the whole, I’m able to deal with stress.	-
I often find it difficult to show my affection to those close to me.	-
I’m normally able to “get into someone’s shoes” and experience their emotions.	-
I normally find it difficult to keep myself motivated.	-
I’m usually able to find ways to control my emotions when I want to.	-
On the whole, I’m pleased with my life.	-
I would describe myself as a good negotiator.	-
I tend to get involved in things I later wish I could get out of.	-
I often pause and think about my feelings.	-
I believe I’m full of personal strengths.	-
I tend to “back down” even if I know I’m right.	-
I don’t seem to have any power at all over other people’s feelings.	-
I generally believe that things will work out fine in my life.	-
I find it difficult to bond well, even with those close to me.	-
Generally, I’m able to adapt to new environments.	-
Others admire me for being relaxed.	-

Appendix B

**Table 9:** Perceived Stress Scale. The Perceived Stress Scale was developed by Cohen et al. (1983) to measure the perceived stress among the participants over the last month [16].

Item	Response
In the last month, how often have you been upset because of something that happened unexpectedly?	-
In the last month, how often have you felt that you were unable to control the important things in your life?	-
In the last month, how often have you felt nervous and stressed?	-
In the last month, how often have you felt confident about your ability to handle your personal problems?	-
In the last month, how often have you felt that things were going your way?	-
In the last month, how often have you found that you could not cope with all the things that you had to do?	-
In the last month, how often have you been able to control irritations in your life?	-
In the last month, how often have you felt that you were on top of things?	-
In the last month, how often have you been angered because of things that happened that were outside of your control?	-
In the last month, how often have you felt difficulties were piling up so high that you could not overcome them?	-

Appendix C

**Table 10:** Job Performance Scale. The Job Performance Scale from Campbell (1990) assess participants’ level of resilience [17].

Items	Response
I am a self-motivated person.	-
I enjoy my work.	-
I am well-trained in my work.	-
I am clear about my duties and responsibilities.	-
I am willing to accept my faults.	-
I receive the respect I deserve from my colleagues.	-
I tend to see problems as challenges rather than as obstacles.	-
The rewards for success are greater than the penalties for failure.	-
The manager encourages me at work.	-
I always receive positive feedback from my employers.	-
Employee Job Performance Working Environment	-
I gain personal growth by learning various skills in my work.	-
The management appreciates my suggestions and leadership.	-
Supervisors encourage me to do well in my work.	-
I am rewarded for the quality of my efforts.	-
I am valued by my supervisor.	-
The company has a positive image towards my friends and family.	-
My job brings positive changes to me.	-
I am able to solve problems immediately to satisfy my manager.	-
I understand the importance to value and respect my colleagues.	-
I am happy with my job.	-
I gain personal accomplishment through my work	-
I have the tools and resources to do my job well.	-
I feel encouraged to come up with new and better ways of doing things.	-
I could clearly define quality goals in my work.	-
My skills and abilities are put into good use in my work.	-
The company does an excellent job in keeping employees informed about matters affecting us.	-
I am satisfied with the information given by the management on what is going on in my division.	-
I am satisfied with my involvement in decisions that affect my work.	-
I feel safe sharing my plans, programs and policies with my management.	-
My manager is committed to finding win-win solutions to problems at work.	-
Employee Job Performance – Salary	-
I am satisfied with my current salary.	-
I am satisfied with my benefit packages.	-
I am satisfied with my most recent increment.	-
I am satisfied with the company’s pay structure.	-
I am satisfied with the amount the company pays my benefits.	-
I am satisfied with the pay raise interval in the company.	-
I am rewarded for the quality of my efforts.	-
I experience personal growth financially in this company.	-
Performance appraisal influences pay raise.	-
There are opportunities for career advancement in this company.	-

Appendix D 

**Table 11:** Resilience Scale. The Resilience Scale of Connor and Davidson (2003) measures the participant’s ability to withstand stress and adversity [18].

Items	Response
Able to adapt to change.	-
Close and secure relationships.	-
Sometimes fate or God can help.	-
Can deal with whatever comes.	-
Past success gives confidence for new challenges.	-
See the humorous side of things.	-
Coping with stress strengthens.	-
Tend to bounce back after illness or hardship.	-
Things happen for a reason.	-
Best effort, no matter what.	-
You can achieve your goals.	-
When things look hopeless, I don’t give up.	-
Know where to turn for help.	-
Under pressure, focus, and think clearly.	-
Prefer to take the lead in problem-solving.	-
Not easily discouraged by failure.	-
Think of yourself as a strong person.	-
Make unpopular or difficult decisions.	-
Can handle unpleasant feelings.	-
Have to act on a hunch.	-
Strong sense of purpose.	-
In control of your life.	-
I like challenges.	-
You work to attain your goals.	-
Pride in your achievements.	-
